# Telerehabilitation Initiated Early in Post-Stroke Recovery: A Feasibility Study

**DOI:** 10.1177/15459683231159660

**Published:** 2023-03-06

**Authors:** Dylan Edwards, Sapna Kumar, Lorie Brinkman, Isabel Cardoso Ferreira, Alberto Esquenazi, Tiffany Nguyen, Michael Su, Stephanie Stein, Jaun May, Allison Hendrix, Casey Finley, Emily Howard, Steven C. Cramer

**Affiliations:** 1 Moss Rehabilitation Research Institute, Elkins Park, PA, USA; 2 UCLA and California Rehabilitation Institute, Los Angeles, CA, USA; 3 MossRehab, Elkins Park, PA, USA

**Keywords:** telerehabilitation, stroke, paresis, motor activity, critical period, upper extremity

## Abstract

**Background:**

Enhanced neural plasticity early after stroke suggests the potential to improve outcomes with intensive rehabilitation therapy. Most patients do not get such therapy, however, due to limited access, changing rehabilitation therapy settings, low therapy doses, and poor compliance.

**Objective:**

To examine the feasibility, safety, and potential efficacy of an established telerehabilitation (TR) program after stroke initiated during admission to an inpatient rehabilitation facility (IRF) and completed in the patient’s home.

**Methods:**

Participants with hemiparetic stroke admitted to an IRF received daily TR targeting arm motor function in addition to usual care. Treatment consisted of 36, 70-minute sessions (half supervised by a licensed therapist via videoconference), over a 6-week period, that included functional games, exercise videos, education, and daily assessments.

**Results:**

Sixteen participants of 19 allocated completed the intervention (age 61.3 ± 9.4 years; 6 female; baseline Upper Extremity Fugl–Meyer [UEFM] score 35.9 ± 6.4 points, mean ± SD; NIHSS score 4 (3.75, 5.25), median, IQR; intervention commenced 28.3 ± 13.0 days post-stroke). Compliance was 100%, retention 84%, and patient satisfaction 93%; 2 patients developed COVID-19 and continued TR. Post-intervention UEFM improvement was 18.1 ± 10.9 points (*P* < .0001); Box and Blocks, 22.4 ± 9.8 blocks (*P* = .0001). Digital motor assessments, acquired daily in the home, were concordant with these gains. The dose of rehabilitation therapy received as usual care during this 6-week interval was 33.9 ± 20.3 hours; adding TR more than doubled this to 73.6 ± 21.8 hours (*P* < .0001). Patients enrolled in Philadelphia could be treated remotely by therapists in Los Angeles.

**Conclusions:**

These results support feasibility, safety, and potential efficacy of providing intense TR therapy early after stroke.

**Clinical Trial Registration:**

clinicaltrials.gov; NCT04657770

## Introduction

Stroke remains a major cause of disability,^1,2^ with hemiparesis being a key contributor. The majority of patients with post-stroke arm weakness demonstrate persistent impairments that are directly linked to larger activity limitations and participation restrictions, lower quality of life, and decreased well-being.^3^ New therapeutic approaches are needed, including strategies to increase overall dose of activity-based motor therapies, that can lead to better outcomes.^4-9^ Telerehabilitation (TR) offers consistent and high therapy doses through greater accessibility, and has the potential to change the landscape for rehabilitation. We previously demonstrated that upper extremity (UE) motor therapy delivered via our telehealth platform improved arm motor status,^10^ and in a multisite, randomized, assessor-blinded, non-inferiority clinical trial, found that this TR program improved arm motor status substantially and to the same extent as dose-matched therapy delivered in the clinic.^11^ This TR therapy further resulted in a significant proportion of patients improving on a global disability scale (modified Rankin Scale [mRS]).^12^ While increasing evidence supports TR in the chronic phase of stroke recovery, data remain scarce for TR started in the early weeks post-stroke, when neural plasticity may be in a heightened state.^13^ Moreover, the transition of rehabilitation care from inpatient rehabilitation to home setting is often fragmented, and outpatient rehabilitation is under-dosed^14^ and delayed in many cases. TR commencing in the Inpatient Rehabilitation Facility (IRF) may enhance the IRF-home transition through continuity of treatment structure and therapist interaction and may also increase the overall therapy dose and compliance during the critical recovery time. The current study aimed to examine feasibility and safety of TR introduced in the IRF setting. The study also aimed to explore UE motor recovery in the setting of early UE motor TR; this was an uncontrolled pilot study, and so this was examined in order to generate an initial estimate of motor outcomes with this therapy. Two secondary goals were to confirm if exercise repetition count during therapy matched the repetition number assigned, and to assess feasibility of having therapists in one city provide TR therapy to patients in a separate city.

## Methods

**Figure 1.**
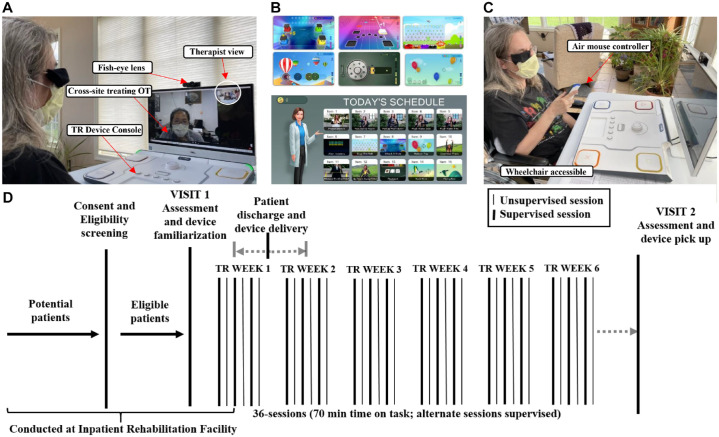
(A) Supervised TR session at home: The in-home setup provides the therapist with a wide-angle view of movements performed by the patient while performing an individualized therapy program, consisting of functional games and exercises. The therapist can also see the screen of the patient’s device (“Therapist view”) and provide feedback. (B) Selected functional games available that target proximal and distal motor functions. These are combined with exercises, assessments, and education into a daily schedule. Patients are presented with a summary of the day’s assigned therapy components and note their progress with each successive component completed. (C) A patient using the air mouse controller, one of several devices available to drive game play, used here improve wrist range of motion during TR game play. (D) Study design: Participants were consented, screened, assessed, and started the TR program while at the IRF, and continued at home with 36 daily sessions of 70-minute duration, 6 days/week, for 6 weeks. Visit 2 was performed within 5 days of the final TR session. Abbreviations: TR, telerehabilitation; IRF, inpatient rehabilitation facility.

This was a prospective, dual-site, single-arm repeated measures study (Figure 1D), approved by the Institutional Review Board at University of California, Los Angeles (which provided oversight for UCLA and MossRehab). We enrolled participants during inpatient admission at California Rehabilitation Institute and MossRehab who had arm weakness after a recent stroke. As this was a pilot study, eligibility criteria were by design broad: age ≥18 years, recent ischemic stroke or intracerebral hemorrhage, at least some UE motor deficits (affected Upper Extremity Fugl–Meyer [UEFM] score <56), and at least some preserved voluntary UE movement (Box and Blocks Test [BBT] ≥3 blocks in 60 seconds); a full list of inclusion/exclusion criteria appears in Table S1. Participant enrollment represented local demographics for a convenience sample without prejudice on sex, race, ethnicity, or socioeconomic status. Stroke admission records to the IRF were screened on a regular basis to identify potential study candidates, who were then approached and, if interested, consented (no surrogate consent). Participants were asked to sign a behavioral contract that outlined study expectations and that also recorded a key functional goal for each patient. Participants were familiarized with their TR system during the IRF admission and then started TR, during the IRF admission when this was possible, depending on length of stay.

A 6-week TR program was implemented, following the intervention protocol from the prior national study,^12^ comprising 36 sessions (18 supervised, 18 unsupervised) of 70-minute length, allowing up to 2 additional weeks for missed sessions. Each 70-minute session began with daily assessment of 4 behaviors: proximal UE movement, via a targeting reaching game by the paretic UE; distal UE movement, assessing maximum tapping speed of the paretic index finger at the metacarpophalangeal joint over 10 taps; general fatigue, using a visual analog scale (VAS); and shoulder pain on the paretic side using a VAS. Each session then involved at least 15 minutes of exercises, at least 15 minutes of functional games, and 5 minutes of stroke education (Figure 1). The TR device includes a range of controllers to provide variety of choice for the therapist promoting specific movements. For example, to focus on gross shoulder and elbow movements, the use of large stationary buttons in the tabletop console, or wireless hand-held controllers may be suitable. For fine movement control, console-based options include a trackpad, a dial, a pinch gage, and small buttons. The treatment intervention centers around high-dose repetitive arm practice; however the overall therapeutic structure includes domains of cognition and attention. For example, games that require UE movement to play might stress visuomotor tracking, memory, or sensorimotor integration. The biological model of TR efficacy is that performance of UE motor repetitions activate numerous brain circuits, including attention, somatosensory, visuomotor, and cognitive circuits, each of which drives motor cortex activity, and the effects of this activity converge on motor cortex and its efferent signals down the corticospinal tract.

Table 1 describes the TR treatment program using the Rehabilitation Treatment Specification System.^15^ The main treatment targets and ingredients promoted by the therapist and by TR system usage include: Organ Functions domain (1) target—increased strength and endurance of UE muscles, ingredient—UE strengthening exercises presented by video, (2) target—increased passive range of motion; ingredient—self-stretching maneuvers using the unaffected hand. Skills and Habits domain (3) target—improved UE motor function; ingredient—digital/visuomotor games with repetitive voluntary gross and fine motor skill practice with extrinsic feedback (including knowledge of performance within-game, and knowledge of results as feedback from scores). Representations domain (4) target—increased cognitive load tolerance, ingredient—progressively increased difficulty in games including speed-accuracy and problem solving, (5) target—transition of device-based practice to ADLs, ingredient—identification of functional goals, modification of TR training to include goals, feedback and review, and (6) target—increased knowledge about stroke and prevention; ingredient—stroke risk factors and preventative measures quiz game with feedback. Each treatment target has a definitive outcome measure. The composite effect of TR treatment aims to have positively ancillary outcomes including adherence to structured daily rehabilitation practice, improved mood, and quality of life.

**Table 1.** Arm Movement Telerehabilitation Description Per the Rehabilitation Treatment Specification System.

Group	Treatment	Target for treatment	Ingredient/s for target
Organ Functions	Adaptive exercise training	Increased strength in proximal to distal muscles of the upper limb: neck, trunk, scapula, shoulder, forearm, elbow, wrist, or hand.	At least 15 min of exercises with each performed for a minimum of 1 min with gravity as resistance, progressed by (1) adding resistance using a weighted dowel, resistance bands, hand gripper, and/or squeeze balls over 6 wk period (2) increasing task demand by adding objects like balls, foam, hand trainer, and dice or blocks in home to simulate components for functional tasks (3) removal of supportive items (progressing for shoulder abduction via pushing PVC pipe, to AROM, to added resistance).
	Stretching	Increased passive range of motion of fingers, wrist, and elbow, and shoulder in the hemiparetic arm	Self-stretching maneuvers using unaffected hand: eg, prayer stretch or clasped hand stretch up and down.
Skills and Habits	Repetitive unilateral arm motor functional practice	Improved arm-hand movement speed, accuracy, and endurance in the hemiparetic arm	At least 15 min of games (digital/visuomotor) involving repetitive voluntary gross and fine motor skill practice with extrinsic feedback due to target practice (knowledge of performance—within the game) and feedback from scores (knowledge of results—end of games and day to day) as well as feedback from clinician on correctness of movement during supervised sessions. Difficulty levels of games were modified to provide the appropriate challenge, based on patient performance (both observed via telehealth platform and on performance statistics provided by the system).
Representations	Stroke education	Increased knowledge of stroke and prevention	Questions on stroke risk factors and preventative measures are displayed in quiz format on the monitor, participants are prompted to select the correct answer and respond performing specific UE movements including using a dial controller and pushing buttons, feedback is provided along with explanation of the fact.
	Functional goals	Identify functional task goals to work on outside of the program practice and work to help transition device-based practice to ADL	Therapist works with the participant to identify functional goals eg, tying shoelaces, typing on a keyboard, etc.; and advises and motivates the participant on ways to practice and improve on the goal. Therapist checks in during supervised sessions to help participant achieve progress toward achieving functional goals. The exercise and game programs were also modified as needed to help reach these goals. Feedback was provided by the therapists on how specific games/exercises directly related to their identified functional goals.
	Progressive load of cognitive demand	Progressively increase cognitive demand required to achieve successful improvement in performance feedback provided by the device during games	The games have different difficulty levels—easy, medium, and hard–that demands increased cognition and problem-solving skills. Therapist makes a judgment based on patient performance to decide the appropriate time to increase difficulty level during the 6-wk program.

Abbreviation: UE, upper extremity.

We documented specific strategies employed in the present study to effectively introduce TR in the IRF setting and adhere to the protocol that may be of use for future trials (Table S2). As in our prior study,^12^ the treatment therapist examined the patient before the first TR therapy session and then created a personalized 70-minute session. This consisted of selecting games and exercises, and adjusting the duration and difficulty of each, as appropriate for each patient’s therapeutic goals. Each patient also performed 5 minutes/day of stroke education, presented in a Jeopardy format. A web-based therapist portal allowed therapists to review patient usage and performance data (including the 4 daily assessments and game scores), plan/modify subsequent day’s treatment sessions, write a brief electronic daily note, and engage in videoconferences during supervised sessions. The remote videoconference set-up allowed flexibility in the location of the supervising therapist, and cross-site supervision between UCLA and MossRehab. During the supervised sessions, therapists checked on individual functional goals and gave the patient feedback as they played games and exercises that targeted specific movements relating to the functional goals.

We tested the feasibility of cross-site treatment supervision and monitoring using 2 models: (1) Full handoff of the patient at IRF discharge. For this model, the interstate therapist from UCLA was introduced to the participant prior to patient discharge from the MossRehab IRF. The baseline assessment scores and insights on patient were shared with the UCLA therapist along with an initial treatment plan drafted by MossRehab to enable smooth transition of care; (2) Shared treatment between local and interstate therapists. As with typical onsite therapy scheduling, for this model, one therapist (interstate) could cover for the principal treatment therapist (local) when a scheduling conflict arises or with time off. For this model, the previous session notes were shared in advance to the covering therapist. The patient was also introduced to the therapist in a 3-way teleconference call prior to the cross-site session to enable effective transition of care.

Adverse events were recorded at supervised sessions. During each supervised session, patients were prompted to report any Adverse Events (AE) that may have occurred since the previous supervised session. AEs were documented in an event reporting form that contained information including: date of event, date the study team learned of the event, 3-tier classification (1. Expected or unexpected, 2. Serious or not serious, 3. Definitely or probably related, possibly related, or unlikely or not related, based on study investigator judgment), plus a description of the event. Clinical outcomes were measured at live exams occurring at baseline and at the end of the intervention. Patient experiences were also collected during and at end of TR using a Telerehab Satisfaction Questionnaire^16^ via the TR system. Two TR hardware systems were employed: the one described previously^12^ and a comparable system manufactured by TRCare (Palo Alto, CA).

Percentage compliance and retention were calculated for study feasibility as the primary outcome measures. Compliance was defined as number of sessions a patient completed with >40 minutes of assigned therapy divided by total number of assigned sessions within 6 to 8 weeks (maximum 36). Retention was defined as % enrollees who completed 15 or more of 18 supervised sessions.^11^ For preliminary estimates of efficacy, clinical outcomes were measured as secondary outcomes, including UEFM, BBT, Montreal Cognitive Assessment (MoCA), 9-hole Peg Test (NHPT), grip strength, pinch strength, 14-item Motor Activity Log, Stroke Impact Scale (SIS), mRS, EuroQol-VAS, Chaos Scale, Modified Ashworth Scale, Shoulder Abduction Finger Extension (SAFE) score, and Trailmaking test A & B. Change over time in these outcome data was assessed using paired-sample parametric or non-parametric tests as appropriate. Change in daily UE motor performance scores representing proximal (whack-a-mole game), and distal (maximum finger tapping speed), pain and fatigue scores were calculated as the difference in average score of the first 3 days and last 3 days.

## Results

**Figure 2.**
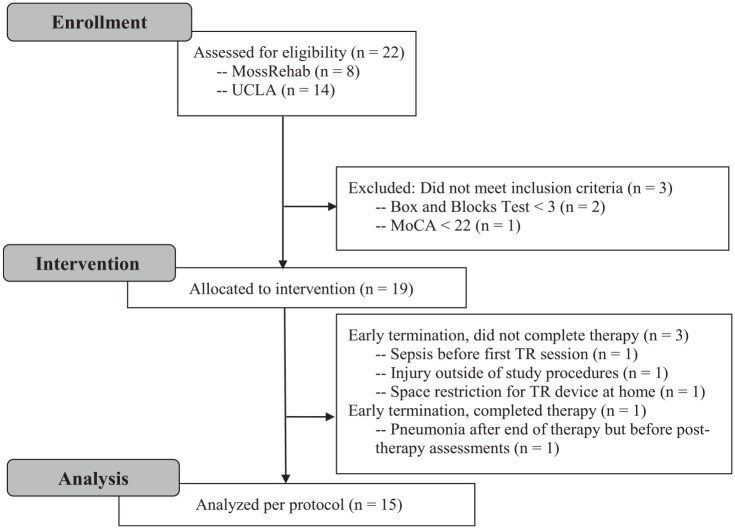
CONSORT flow diagram. Abbraviations: MoCA, Montreal Cognitive Assessment; TR, telerehabilitation.

From 5/13/2021 to 5/6/2022, 831 admission records between the 2 sites were pre-screened for potential study eligibility. Of 63 records indicating potential eligibility, 22 participants were both approached and gave written informed consent to participate. 19 remained eligible after in-person screening for inclusion and were allocated to the intervention. 16 completed the intervention (84.2% retention), and 15 participants additionally completed the post-intervention visit (Figure 2). The three participants who dropped out had comparable baseline clinical features (UEFM, BBT, MoCA, Geriatric Depression Scale, *P* > .05), and although they were significantly younger as a group (51.2 ± 4.0 years, dropout; 63.4 ± 11.5, completed; *P* = .04), the cause of withdrawal (Figure 2) is unlikely to be attributable to age.

The baseline characteristics of the 16 patients who completed the intervention are presented in Table 2. Time from stroke to consent was 20.1 ± 12.0 (mean ± SD) days; to treatment day 1, 28.3 ± 13.0 days (range 10-71). The 16 participants who completed the intervention had overall low global impairment (NIHSS score 4 [3.75, 5.25], median, IQR), mild-to-no aphasia, and moderate-severe hemiparesis (UEFM score 35.9 ± 6.4 points). The number of TR sessions completed within the IRF was 4.4 ± 4.4 (range 0-13). Compliance with the 36 TR sessions was 100% (97%, 100%). Note that 2 patients had a new COVID-19 diagnosis during enrollment and continued TR. Compliance was not affected with cross-site treatment supervision and monitoring, irrespective of whether the treatment was shared between local and interstate therapists, or fully transitioned to the interstate therapist. Patient acceptance of the program, measured using percentage maximum score on the Telerehab Satisfaction Questionnaire, had a median value of 93.3% (IQR 82%-95%). The dose of rehabilitation therapy for usual care during this 6-week interval was 33.9 ± 20.3 hours. This comprised both inpatient and outpatient rehabilitation therapy (usual care data available in 11 subjects). The total dose of rehabilitation therapy more than doubled (*P* < .0001), to 73.6 ± 21.8 hours, when the amount of TR (received in IRF and then at home) was added.

**Table 2.** Baseline Characteristics.

A. Demographics and medical information		B. Baseline clinical assessments (non-motor)		C. Baseline clinical assessments (motor)	
Age, mean (SD)	61.26 (±9.36)	NIH Stroke Scale (median) [IQR]	4 [3.75, 5.25]	UE Fugl–Meyer (total)	35.88 (±6.40)
Sex, n (%)		mRS	3 [2, 4]	Proximal	21.19 (±4.94)
Male	10 (62.5%)	SIS		Wrist/Hand	11.75 (±4.58)
Female	6 (37.5%)	ADL section	32.26 (±6.68)	Coordination/speed	2.94 (±1.0)
Race, n (%)		Hand motor domain	13.58 (±5.17)	Handedness	R = 11, A = 2, L = 3
American Indian or Alaska Native	0 (0%)	Geriatric Depression Scale	3.75 (±3.38)	14 item motor activity log	16.13 (±12.04)
Asian	4 (25%)	EQ VAS	71.10 (±17.31)	SAFE	6.44 (±1.63)
Black or African American	6 (37.5%)	Chaos scale	14.17 (±4.06)	BBT (# of blocks)	
Native Hawaiian/Pacific Islander	0 (0%)	MoCA	24.69 (±3.98)	Affected UE	13 (±11.16)
White	6 (37.5%)	Line cancellation test, n (%)		Unaffected UE	40.13 (±12.30)
Unknown	0 (0%)	Normal	14 (87.5%)	Grip strength (kg)	
Ethnicity, n (%)		Abnormal	2 (12.5%)	Affected UE	8.82 (±6.97)
Hispanic/Latino	0 (0%)	Trail Making Test		Unaffected UE	28.74 (±9.96)
Not Hispanic/Latino	15 (93.8%)	Time in sec		Pinch strength (kg)	
Unknown	1 (6.3%)	Test A	45.22 (±18.48)	Pinch strength (affected) kg	3.24 (±3.26)
Comorbidities, n (%)		Test B	113.31 (±55.37)	Pinch strength (unaffected) kg	7.83 (±3.15)
Hypertension	10 (62.5%)	Correct connections		Modified Ashworth Scale	
High cholesterol	8 (50%)	Test A	23.94 (±3.49)	Pectoralis Major	0 [0, 1]
Diabetes mellitus	2 (12.5%)	Test B	18.25 (±7.36)	Biceps	1 [.5, 1.5]
Atrial fibrillation	1 (6.25%)	Nottingham sensory scale		Triceps	0 [0, 1]
Coronary artery disease	2 (12.5%)	Light touch	3.19 (±1.52)	Wrist Flexors	0 [0, 1]
Prior stroke, n (%)		Pin Prick	3.31 (±1.01)	Finger Flexors	0 [0, 1]
No prior stroke	14 (87.5%)	Kinesthesia	8.50 (±2.92)	NHPT	
≥1 prior stroke (with remaining motor symptoms)	2 (12.5%)	Stereognosis	10.25 (±9.90)	Affected UE	
Index stroke				Time to complete	55.91 (±10.96)
Lesion type, n (%)				Pegs placed (of 9)	3.40 (±3.56)
Ischemic	13 (81.3%)			Pegs removed (of 9)	1.33 (±3.15)
Hemorrhagic	3 (18.8%)			Unaffected UE	
Time since stroke (stroke to consent)				Time to complete	35.75 (±16.98)
Mean days (±SD)	20.13 (±12.02)			Pegs placed (of 9)	7.60 (±2.75)
<14 d, n (%)	3 (18.75%)			Pegs removed (of 9)	8.25 (±2.60)
14-30 d, n (%)	11 (68.75%)				
>30 d, n (%)	2 (12.5%)				

Abbreviations: UE, upper extremity; mRS, modified Rankin Scale; SIS, Stroke Impact Scale; SAFE, Shoulder Abduction Finger Extension; BBT, Box and Blocks Test; MoCA, Montreal Cognitive Assessment; NHPT, 9-Hole Peg Test.

Behavioral outcomes support the potential efficacy of this TR intervention. Change in behavioral scores from baseline to immediately post-TR are listed in Table 3 (n = 15). Patients receiving TR showed good recovery: post-intervention, UEFM improvement was 18.1 ± 10.9 points (*P* = .0001, MCID 5.25pts^17^), BBT improvement was 22.4 ± 9.9 blocks (*P* = .0001), 9-hole peg test completion time decreased by 17.3 ± 12.8 seconds (*P* = .004), and Motor Activity Log increased by 24.0 ± 13.8 points (*P* = .0001). Additional motor tests that improved significantly (*P* < .05) included the SAFE score, SIS hand motor domain, and pinch strength. The mRS score improved by 1 point (median −1; IQR −2, 0; *P* = .004) from pre- to post-intervention.

**Table 3.** Clinical Change From Baseline to Immediately After Completing Telerehabilitation Therapy.

Assessment	Pre-intervention score	Post-intervention score	Change scores	*P*
Geriatric Depression Scale mean (SD)	3.87 (±3.46)	4.36 (±4.13)	0.2 ± 4.38	.95
SIS (ADL section)	30.27 (±5.60)	34.71 (±7.53)	2.13 ± 9.99	.11
Stroke impact scale (hand motor domain)	10.47(±4.45)	16.57 (±4.24)	5 ± 3.67	.**0006**
EQ VAS	69.87 (±21.04)	74.21 (±13.35)	−0.6 ± 24.87	.95
Chaos scale	14.27 (±3.83)	13.93 (±4.55)	−1.27 ± 3.15	.23
mRS (median) [IQR]	4 [3, 4]	2 [2, 3]	−1 [−2, 0]	.**0039**
Modified Ashworth Scale				
Pectoralis Major	0 [0, 1.5]	0 [0, 1]	0 [−0.5, 1]	1
Biceps	1 [1, 1.5]	1 [0, 2]	0 [−0.5, 0.5]	.82
Triceps	0 [0, 1]	0 [0, 1.5]	0 [−0.5, 0.5]	.95
Wrist Flexors	0 [0, 1]	0 [0, 1]	0 [−0.5, 0]	.84
Finger Flexors	0 [0, 1]	1 [0, 1]	0 [0, 1]	.31
14 item motor activity log	17.20 (±11.91)	41.23 (±10.30)	24.03 ± 13.81	.**0001**
SAFE	6.33 (±1.63)	8.27 (±1.16)	1.98 ± 1.81	.**002**
Grip strength (affected) kg	8.78 (±7.21)	15.14 (±6.86)	6.35 ± 9.39	.**081**
Grip strength (unaffected) kg	29.50 (±9.82)	29.29 (±9.19)	−0.21 ± 4.8	.72
Pinch strength (affected) kg	3.26 (±3.40)	5.67 (±3.00)	2.4 ± 1.93	.**0009**
Pinch strength (unaffected) kg	8.11 (±3.05)	8.32 (±3.93)	0.2 ± 1.38	.77
MoCA	24.73 (±4.11)	26.27 (±3.37)	1.53 ± 3.12	.11
BBT (affected) # of blocks in 60 s	11.53 (±9.83)	33.93 (±10.74)	22.4 ± 9.75	.**0001**
BBT (unaffected) # of blocks in 60 s	39.60 (±12.54)	44.20 (±12.58)	4.76 ± 8.33	.07
Fugl–Meyer Assessment (total)	35.33 (±6.23)	53.40 (±9.26)	18.07 ± 10.85	**<.0001**
Fugl–Meyer Assessment (proximal)	20.60 (±4.50)	28.80 (±5.77)	8.2 ± 5.98	.**0001**
Fugl–Meyer Assessment (Wrist/Hand)	11.67 (±4.73)	19.87 (±3.29)	8.2 ± 5.9	.**0001**
Fugl–Meyer Assessment (coordination/speed)	3.07 (±0.88)	4.73 (±1.10)	1.67 ± 1.01	.**0002**
Trail Making Test				
Test A				
Time in sec	43.70 (±18.10)	32.84 (±8.59)	−10.86 ± 18.65	.**03**
Correct connections (of 25)	23.87 (±3.48)	23.73 (±4.48)	−0.13 ± 1.15	1
Test B				
Time in sec	113.47 (±57.18)	125.37 (±70.55)	11.9 ± 54.29	.81
Correct connections (of 25)	19.20 (±6.58)	17.93 (±8.19)	−1.27 ± 6.87	.79
NHPT				
NHPT affected time to complete (s)	57.85 (±7.45)	40.52 (±14.78)	−17.33 ± 12.75	.**004**
NHPT affected pegs placed (of 9)	2.83 (±3.59)	8.08 (±2.61)	5.25 ± 3.57	.**0039**
NHPT affected pegs removed (of 9)	0.920(±2.61)	6.75 (±4.07)	5.83 ± 4.16	.**0078**
NHPT unaffected time to complete (s)	29.68 (±12.89)	28.33 (±12.39)	−1.36 ± 9.41	.79
NHPT unaffected pegs placed (of 9)	8.58 (±1.44)	8.42 (±2.02)	−0.17 ± 0.55	1
NHPT unaffected pegs removed (of 9)	8.25 (±2.60)	8.25 (±2.60)	0 ± 0	1

Abbreviations: SIS, Stroke Impact Scale; mRS, modified Rankin Scale; MoCA, Montreal Cognitive Assessment; BBT, Box and Blocks Test; NHPT, 9-hole Peg Test.

*P* values in bold indicate statistical significance.

**Figure 3.**
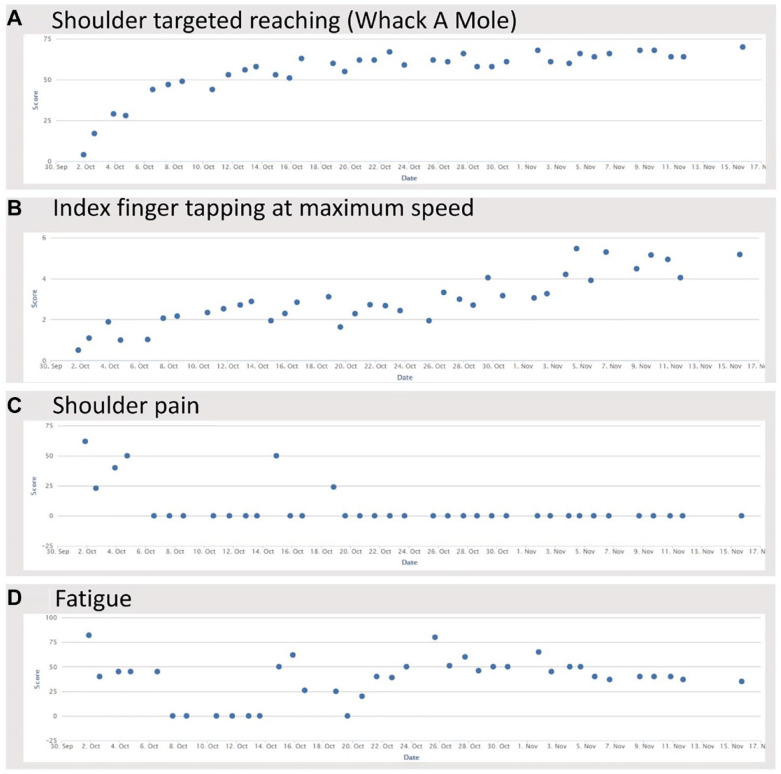
Each patient underwent 4 daily assessments using the telerehabilitation system. Sample data from an individual participant are shown, with improvement over time in (A) proximal motor performance, (B) distal motor performance, and (C) shoulder pain. Panel (D) shows fatigue to be stable. Index finger tapping is reported in Hz.

Daily assessments of UE motor status, fatigue, and shoulder pain could be collected using the TR system from the IRF and then from the home. Daily motor scores improved over the 36 treatment sessions: the proximal UE task increased from 30.0 ± 15.2 to 69.9 ± 26.1 targets (*P* = .0005) and maximum index finger tapping rate increased from 0.73 ± 0.42 to 3.0 ± 1.3 Hz (*P* = .0005; n = 12). Daily pain and fatigue scores also improved, but these changes did not reach significance. An example of daily testing in a patient appears in Figure 3.

The number of UE repetitions for assigned exercises agreed closely with a measure based on a therapist counting movements during therapy across the 18 supervised sessions (assigned repetitions = 3881 ± 1805; therapist count = 4024 ± 1642; Spearman’s rho = .98, *P* < .0001).

A total of 6 AEs were recorded: 4 were classified as unexpected, serious, and unrelated to the study—sepsis, pneumonia, recurrent stroke, and shoulder injury outside of study; 2 were classified as unexpected, not serious, possibly related—a patient with hemianopsia felt nausea when using his prism glasses during TR (relieved by increasing breaks) and a patient with depression felt demotivated when asked to score anxiety and depression online (relieved by removing these assessments).

## Discussion

This study demonstrated the feasibility of providing intensive TR-based arm therapy in addition to usual care, starting during IRF admission and at IRF discharge continuing seamlessly in the home, for a regimen spanning 6 weeks. The addition of TR substantially increased the overall therapy dose. Treatment on one side of the continent could be supervised by a therapist on the opposite side, supporting that TR treatment need not be limited to the catchment area of a local hospital.

### Feasibility Demonstrated: TR Can Start in the IRF and Transition to the Home

A key question for intensive add-on therapies in the early subacute period after stroke is whether participants can be identified, consented, assessed, and then commence treatment whilst admitted to the IRF. Our current findings support this—all within a month from stroke onset on average. All patients completed familiarization sessions within the IRF and received up to 13 TR therapy sessions in addition to usual therapy before transitioning the TR therapy to home. This was in conjunction with all usual IRF care, and without significant disruption to clinical workflow. Patients commenced TR therapy as early as 10 days after stroke onset. Adding TR more than doubled the total dose of therapy provided by usual care alone during the subacute period post-stroke. Furthermore, we validated that the assigned number of UE movement repetitions during exercises closely matched the number observed by therapists during supervised sessions.

Adherence to the TR exercise regimen was excellent (median 100%), markedly higher than the previously reported 65% in post-stroke rehabilitation.^18^ This technology spans the transition from IRF to home, employing the same device, treatment content, and treatment therapist over time, which may explain the high compliance and satisfaction. Because TR continued in the home, two patients with a new COVID-19 diagnosis and isolation could continue post-stroke rehabilitation therapy. Anecdotally, these factors were valued by patients and are consistent with the high (93%) patient satisfaction scores at the end of TR. One patient reported TR-home-therapy as “the single most important thing each day that gets me out of bed in the morning.”

### Potential Efficacy and Low Risk of Early TR Intervention

Significantly improved clinical scores were observed across a range of measures, although the contribution of TR is difficult to discern given the lack of a control group and the expected natural recovery in the early post stroke phase.^8^ Future studies should evaluate the effect of adding TR to usual care early post-stroke compared to a control group receiving usual care only in order to define benefits specifically attributable to adding TR therapy to usual care.

There were no serious adverse events related to the intervention despite the increased overall dose of motor activity, and minor events could be resolved without impacting therapy. This finding is in alignment with the US Food and Drug Administration designation of the TR device and protocol as non-significant risk. The several unrelated serious adverse events represent general medical complications that are prevalent in early recovery. In this patient population, medical complications in some cases may delay or interrupt the TR intervention, but are less likely to result in the patient being excluded from this therapy. No evidence of harmful effects of this early and intensive intervention was apparent, but rather association with large clinical improvements. Our interpretation is that the potential benefit of this accessible intervention outweighs the negligible risk.

### Patient Suitability for TR and Optimal Timing

For which patients might the TR intervention be suitable? Similar to conventional motor therapies that do not use digital technology, this type of TR intervention should be suitable for stroke patients with persistent arm motor impairment who have sufficient residual motor and cognitive ability to engage in repetitive movements and therefore might be reasonably expected to benefit. Our study inclusion criteria were broad in some respects, enabling patients with severely reduced function to participate (minimum 3 blocks per minute, BBT), and thus enrollment was not restricted to high functioning patients. Patients with a mild decrease in mental status (MoCA >21) could participate, and those with mild-moderate forms of common coexisting conditions such as depression and aphasia could also take part. Since reduced arm function is common amongst patients with stroke, the significance of a treatment that may enhance arm function^11^ and reduce global disability^12^ should be underscored.

Relative to historic clinical trials in post stroke motor recovery, the pre-screening hit-rate for the present study would not be considered outside a typical range.^9,19,20^ We did not find that the nature of our intervention being digitally based and involving remotely supervised sessions limited the enrollment or selection of patients. Indeed, the high compliance rate with TR observed following enrollment may actually be attributable in part to the nature of our TR intervention, for example, through strong engagement and connection with the therapist.

Young patient age and high computer literacy do not likely explain this study’s successful enrollment and high compliance. The mean age of adult participants in the present study was 61 ± 9 years, comparable to that seen in activity-based motor studies in adult stroke that do not use such technology.^9,19^ We previously reported that although computer literacy declined with increasing age as expected, motor gains and amount of TR system use were not affected by computer literacy.^10^ Thus, we maintain that our system is easy to use, does not require computer skills, and is suitable for the adult stroke population.

The prediction of individual patients achieving a clinically meaningful improvement from TR was not a focus of the present study. As with conventional activity-based motor rehabilitation, patient selection and outcome prediction is challenging using baseline clinical data alone. In one recent study, we found that a model combining multiple clinical measures explained only 39.3% of the variance in treatment-related gains following TR in patients with stroke.^21^ We expect that prediction would be strengthened by incorporating a measure of integrity of the ipsilesional corticospinal tract.^22-26^ The biological reserve (% residual CST) is a leading criterion for positive intervention response,^25^ and may outperform judgment based on clinical features. This would be useful to assess in prospective trials, and has practical utility with routine clinical MRI acquisition progressively more common in stroke diagnosis.

We have demonstrated that our TR intervention previously shown to be effective in the late subacute to chronic stage, is now feasible and safe in early recovery at less than 1 month from stroke onset. Early intensive intervention that spans from the IRF setting to the home/community setting is a practical solution for addressing the under-dosing inherent in contemporary clinical care in this recovery phase. It is also considered a critical time for brain plasticity, with potential to alter the recovery trajectory. Our findings here set the stage for a trial comparing TR intervention commencing in the early sub-acute versus late sub-acute recovery phases. The outcome would determine if the TR should start in the IRF, or can be (or should be) commenced later in the community setting, an open question.

### Study Limitations

As an initial feasibility study of early intensive TR, the present study was not designed to include a control group, and so the clinical improvement data presented here should be interpreted in this light. Future efficacy trials of this intervention should include a usual care control group, to enable the clinical improvement with usual care + TR, to be contrasted against that of usual care alone, in order to examine the efficacy of the TR intervention in early recovery after stroke.

The choice of outcome measures in the present study was concordant with treatment content, that is, arm motor impairment and function. While UE impairment is most often measured using the UEFM, the outcome measure of choice for arm function is debated. For our preliminary assessment of functional efficacy here, we used the BBT to quantify arm function and NHPT for distal function, due to their historical acceptance, and being relatively unaffected by ceiling effects. Future studies might also consider the Action Research Arm Test, an accepted and standardized test of arm function,^3,27,28^ that would enable results interpretation in the context of similar recovery clinical trials such as the recent CPASS trial.^9^

## Conclusions

The present study demonstrates the safety, feasibility, and favorable rating of intensive arm rehabilitation using a digital telehealth platform within the U.S. IRF setting, over and above usual rehabilitation care. The intervention was effectively transitioned from IRF to the home environment, where patients continued with high compliance and satisfaction. The dose of therapy when adding TR more than doubled what was provided by usual care.

## Supplemental Material

sj-docx-1-nnr-10.1177_15459683231159660 – Supplemental material for Telerehabilitation Initiated Early in Post-Stroke Recovery: A Feasibility Study Click here for additional data file. Supplemental material, sj-docx-1-nnr-10.1177_15459683231159660 for Telerehabilitation Initiated Early in Post-Stroke Recovery: A Feasibility Study by Dylan Edwards, Sapna Kumar, Lorie Brinkman, Isabel Cardoso Ferreira, Alberto Esquenazi, Tiffany Nguyen, Michael Su, Stephanie Stein, Jaun May, Allison Hendrix, Casey Finley, Emily Howard and Steven C. Cramer in Neurorehabilitation and Neural Repair

## References

[1] Tsao CW Aday AW Almarzooq ZI , et al. Heart disease and stroke statistics-2022 update: a report from the American Heart Association. Circulation. 2022;145(8):e153-e639. doi:10.1161/CIR.0000000000001052 35078371

[2] Ovbiagele B Goldstein LB Higashida RT , et al. Forecasting the future of stroke in the United States: a policy statement from the American Heart Association and American Stroke Association. Stroke. 2013;44:2361-2375. doi:10.1161/STR.0b013e31829734f2 23697546

[3] Winstein CJ Stein J Arena R , et al. Guidelines for adult stroke rehabilitation and recovery: a guideline for healthcare professionals from the American Heart Association/American Stroke Association. Stroke. 2016;47(6):e98-e169. doi:10.1161/STR.0000000000000098 27145936

[4] Kwakkel G Van Peppen R Wagenaar RC , et al. Effects of augmented exercise therapy time after stroke: a meta-analysis. Stroke. 2004;35:2529-2539. doi:10.1161/01.STR.0000143153.76460.7d 15472114

[5] Lohse KR Lang CE Boyd LA . Is more better? Using metadata to explore dose-response relationships in stroke rehabilitation. Stroke. 2014;45(7):2053-2058. doi:10.1161/STROKEAHA.114.004695 24867924 PMC4071164

[6] Schneider EJ Lannin NA Ada L Schmidt J . Increasing the amount of usual rehabilitation improves activity after stroke: a systematic review. J Physiother. 2016;62(4):182-187. doi:10.1016/j.jphys.2016.08.006 27637769

[7] Winstein C Kim B Kim S Martinez C Schweighofer N . Dosage matters. Stroke. 2019;50(7):1831-1837. doi:10.1161/strokeaha.118.023603 31164067 PMC12718071

[8] Esquenazi A Lee S Watanabe T , et al. A comparison of the armeo to tabletop-assisted therapy exercises as supplemental interventions in acute stroke rehabilitation: a randomized single blind study. PM R. 2021;13(1):30-37. doi:10.1002/pmrj.12397 32358874

[9] Dromerick AW Geed S Barth J , et al. Critical Period After Stroke Study (CPASS): a phase II clinical trial testing an optimal time for motor recovery after stroke in humans. Proc Natl Acad Sci USA. 2021;118(39):e2026676118. doi:10.1073/pnas.2026676118 PMC8488696 34544853

[10] Dodakian L McKenzie AL Le V , et al. A home-based telerehabilitation program for patients with stroke. Neurorehabil Neural Repair. 2017;31:923-933. doi:10.1177/1545968317733818 29072556 PMC5734923

[11] Cramer SC Dodakian L Le V , et al. Efficacy of home-based telerehabilitation vs in-clinic therapy for adults after stroke: a randomized clinical trial. JAMA Neurol. 2019;76(9):1079-1087. doi:10.1001/jamaneurol.2019.1604 31233135 PMC6593624

[12] Cramer SC Le V Saver JL , et al. Intense arm rehabilitation therapy improves the modified rankin scale score: association between gains in impairment and function. Neurology. 2021;96(14):e1812-e1822. doi:10.1212/WNL.0000000000011667 PMC8105969 33589538

[13] Biernaskie J Chernenko G Corbett D . Efficacy of rehabilitative experience declines with time after focal ischemic brain injury. J Neurosci. 2004;24(5):1245-1254. doi:10.1523/JNEUROSCI.3834-03.2004 14762143 PMC6793570

[14] Lang CE MacDonald JR Reisman DS , et al. Observation of amounts of movement practice provided during stroke rehabilitation. Arch Phys Med Rehabil. 2009;90(10):1692-1698. doi:10.1016/j.apmr.2009.04.005 19801058 PMC3008558

[15] Van Stan JH Whyte J Duffy JR , et al. Rehabilitation treatment specification system: methodology to identify and describe unique targets and ingredients. Arch Phys Med Rehabil. 2021;102(3):521-531. doi:10.1016/j.apmr.2020.09.383 33065124 PMC7934085

[16] Piron L Turolla A Tonin P Piccione F Lain L Dam M . Satisfaction with care in post-stroke patients undergoing a telerehabilitation programme at home. J Telemed Telecare. 2008;14(5):257-260. doi:10.1258/jtt.2008.080304 18633001

[17] Page SJ Fulk GD Boyne P . Clinically important differences for the upper-extremity Fugl-Meyer scale in people with minimal to moderate impairment due to chronic stroke. Phys Ther. 2012;92:791-798. doi:10.2522/ptj.20110009 22282773

[18] Miller KK Porter RE DeBaun-Sprague E Van Puymbroeck M Schmid AA . Exercise after stroke: patient adherence and beliefs after discharge from rehabilitation. Top Stroke Rehabil. 2017;24(2):142-148. doi:10.1080/10749357.2016.1200292 27334684

[19] Winstein CJ Wolf SL Dromerick AW , et al. Effect of a task-oriented rehabilitation program on upper extremity recovery following motor stroke the ICARE randomized clinical trial. JAMA. 2016;315:571-581. doi:10.1001/jama.2016.0276 26864411 PMC4795962

[20] Dromerick AW Lang CE Birkenmeier RL , et al. Very early constraint-induced movement during stroke rehabilitation (VECTORS): a single-center RCT. Neurology. 2009;73(3):195-201. doi:10.1212/WNL.0b013e3181ab2b27 19458319 PMC2715572

[21] Paik SM Cramer SC . Predicting motor gains with home-based telerehabilitation after stroke. J Telemed Telecare. Published online June 22, 2021. doi:10.1177/1357633X211023353 34156873

[22] Lin DJ Cloutier AM Erler KS , et al. Corticospinal tract injury estimated from acute stroke imaging predicts upper extremity motor recovery after stroke. Stroke. 2019;50(12):3569-3577. doi:10.1161/STROKEAHA.119.025898 31648631 PMC6878199

[23] Riley JD Le V Der-Yeghiaian L , et al. Anatomy of stroke injury predicts gains from therapy. Stroke. 2011;42(2):421-426. doi:10.1161/STROKEAHA.110.599340 21164128 PMC3026869

[24] Nouri S Cramer SC . Anatomy and physiology predict response to motor cortex stimulation after stroke. Neurology. 2011;77(11):1076-1083. doi:10.1212/WNL.0b013e31822e1482 21880996 PMC3265049

[25] Cassidy JM Tran G Quinlan EB Cramer SC . Neuroimaging identifies patients most likely to respond to a restorative stroke therapy. Stroke. 2018;49(2):433-438. doi:10.1161/STROKEAHA.117.018844 29321336 PMC5780222

[26] Quinlan EB Dodakian L See J , et al. Neural function, injury, and stroke subtype predict treatment gains after stroke. Ann Neurol. 2015;77(1):132-145. doi:10.1002/ana.24309 25382315 PMC4293339

[27] Yozbatiran N Der-Yeghiaian L Cramer SC . A standardized approach to performing the action research arm test. Neurorehabil Neural Repair. 2008;22(1):78-90. doi:10.1177/1545968307305353 17704352

[28] Lang CE Wagner JM Dromerick AW Edwards DF . Measurement of upper-extremity function early after stroke: properties of the action research arm test. Arch Phys Med Rehabil. 2006;87(12):1605-1610. doi:10.1016/j.apmr.2006.09.003 17141640

