# Supplementary material for: Telerehabilitation Initiated Early in Post-Stroke Recovery: A Feasibility Study
Source: Neurorehabil Neural Repair. 2023 Mar 6;37(2-3):131–41. doi: 10.1177/15459683231159660 (PMC10080366; doi:10.1177/15459683231159660)
Supplement: sj-docx-1-nnr-10.1177_15459683231159660 – Supplemental material for Telerehabilitation Initiated Early in Post-Stroke Recovery: A Feasibility Study [file sj-docx-1-nnr-10.1177_15459683231159660.docx]

ONLINE SUPPLEMENTARY MATERIAL

Telerehabilitation initiated in early post-stroke recovery: A feasibility study

**Table S1. Eligibility criteria**

Inclusion Criteria

- Age 18 years or older
- Stroke that has been radiologically verified (ischemic or intracerebral hemorrhage)
- UEFM score <56 (out of 66)
- Box and Blocks Test (BBT) score ≥3 blocks
- Able to sign informed consent and behavioral contract
- Admitted to California Rehabilitation Institute or MossRehab for stroke rehabilitation

Exclusion Criteria

- Major, active, coexistent neurological or psychiatric disease (e.g., alcoholism or dementia)
- Major medical comorbidities that reduces subject’s ability to comply with study procedures
- Severe depression (Geriatric Depression Scale Score >10/15)
- Significant cognitive impairment (MoCA <22) (Lower score permitted if due to aphasia and PI permits)
- Communication deficits interfering with reasonable study participation
- Decreased visual acuity with/without corrective lens (must be ≥20/40 in at least one eye)
- Life expectancy <6 months
- Pregnant
- Botulinum toxin to in preceding 6 months or expected to receive botulinum toxin within 3 months of study enrollment
- Unable to successfully perform all 3 of the rehabilitation exercise test examples
- Unable or unwilling to perform study procedures/therapy or attend study visits, or expectation of noncompliance with study procedures/therapy
- Non-English speaking – does not speak sufficient English to comply with study procedures
- Will not have single domicile address during 6 weeks of therapy that (1) has Verizon wireless reception or a home WiFi network and (2) has space for TR system
- Has symptoms or diagnosis of COVID-19 during 21 days prior to enrollment.

**Table S2. Telerehabilitation Implementation and Compliance Recommendations**

| **Recommendations** | **Examples** |
| --- | --- |
| **I. At the IRF** | |
| **A. General Fatigue - physical/mental** | |
| Flexibility in scheduling for Inpatient United States Rehabilitation 3-hour compliance requirement  1) Assessments may be done during the day with flexibility to break up into multiple sessions or reschedule on inpatient staff request.  2) Telerehabilitation training sessions scheduled at or after 3.30pm and end before patient's dinner time with flexibility to reschedule.   3) Allow patients to split the sessions throughout the day. | We had initially scheduled an assessment session for a participant on a morning and after request from inpatient OT to reschedule due to staffing rearrangements, we rescheduled the session to a different time of the day and had a backup session scheduled for the following day. |
| **B. Speed of learning (due to age, computer experience/familiarization, cognitive deficits)** | |
| 1) Consider including caregiver (in-person or over a video call) during familiarization sessions. Highly recommend caregiver engagement for such participants.  2) Provide up to 3 familiarization sessions* (covering device introduction; use of input devices; acquaintance to games and exercises, stroke education and assessments, use of video conferencing system etc.).   3) Enroll patients closer to admission rather than discharge to provide ample time for familiarization and practice.  4) Frontload supervised sessions during time at IRF, if needed **Consider optional additional familiarization sessions for some patients as and when needed, at the judgement of the clinician.* | 1) We had a participant who had memory issues and required more familiarization sessions. We invited the caregiver to join one of the familiarization sessions, since she was going to be pivotal in helping him during unsupervised sessions  2) We had enrolled a participant 3 days after admission who had multiple rescheduling and a recurrent stroke. In spite of the unanticipated events, we were able to complete all assessment and familiarization sessions and keep him enrolled in the program |
| **C. Existing comorbidities and new medical conditions (e.g., recurrent stroke during IRF, or COVID-19 infection)** | |
| 1) Obtain Attending physician's opinion on patient's suitability for study prior to approaching patient, where one can bring up any medical conditions/personal situations that may not have been evidently documented in their electronic medical record.  2) Rescreening and redoing eligibility assessments in case of recurrent stroke/ onset of any new diagnosis after consenting to the study. | 1) One of our participants had neuropsychological issues that were not evident from chart review. The attending physician had recommended we check in with his neuropsychologist before we approached him. After approaching, the patient considered the study initially and declined later due to anxiety issues which both the attending physician and neuropsychologist had brought up.   2) We enrolled a participant 3 days after admission who had multiple rescheduling and a recurrent stroke. We were able to complete all re-assessments and familiarization sessions and keep him enrolled in the program. |
| Note: If commencement of the 36-session regimen at IRF is prohibitive due to hindrance of inpatient schedule, reduce telerehab goals during IRF admission to become familiarization with the TR system, then start TR therapy sessions after discharge from the IRF. | |
| **II. At home- Patient extrinsic factors** | |
| **A. Rare technological issues** | |
| 1) Support from caregiver to help with the starting the software used by patients at home to join a videoconference on supervised sessions.  2) On-call tech support: A therapist or research assistant was available to remote-in to patient’s home-based TR computer to help with any tech issues that might arise, e.g., joining video conference on supervised sessions.  3) Staff access to a spare TR system in order to be able to provide accurate instruction to patient/caregiver during troubleshooting process (especially in case of occasional internet connectivity issues). | 1) Our first participant had her daughter (a caregiver) help her with some troubleshooting issues for the first week before the participant could get used to performing TR steps herself in cases such as videoconferencing connectivity issues.   2) A research assistant was always available in any case where remote access software was not connecting for the therapist, to remotely connect with the patient’s home-based TR computer in order to help the patient join a videoconference call |
| **B. Device issues** | |
| 1) Tech support: A therapist or research assistant was available to remotely connect to the patient’s home-based TR computer to help with any TR device performance issues.   2) A research assistant was available to visit the patient's home to troubleshoot any hardware issues that could not be resolved remotely, e.g., to replace dead batteries or plug back in a cable that became disconnected.   3) Availability of back up equipment for replacement in case of hardware failure. | 1) One of our participants had trouble with the "X" button not working and the console not staying on. RA was able to bring a spare console to the patient’s home and fixed the issue. |
| **C. Unexpected trips/ family issues** | |
| Principal Investigator or responsible clinician should call patient to check in and conduct a survey to identify reason for non-compliance after patient misses 2 or more sessions; remind about the behavioral contract signed at the beginning of the study; repeat as needed if patient misses 2 more sessions after the check in call. | Site PI checked in with 2 patients who had a brief interruption in therapy to help them return to their TR program. |
| **III. At home (Unsupervised) - Patient intrinsic factors** | |
| **A. Lack of motivation** | |
| In cases where patient is considered at risk of low compliance, reminder messages may be displayed on the device screen regarding date and time of next session; If concerns remain, reminder calls/text from the therapist or research assistant may be employed. | One of our participants was only complying with supervised sessions and was not completing unsupervised sessions. A check in phone call from the site Principal Investigator, monitoring of performance log by the RA, and text message reminders helped her complete unsupervised sessions. |
| **B. Lack of awareness of unsupervised session monitoring** | |
| Behavioral contract to include text mentioning that the unsupervised session activities are monitored closely, and the study team can remove the patient from the study if patient misses or fails to complete 11 or more scheduled sessions. (supervised and/or unsupervised) | The PI identified that a participant who was not completing unsupervised sessions was not aware of the fact that the therapist or research assistant can monitor her performance from the portal even on unsupervised days. Stating this explicitly in the behavioral contract would act as a reminder. |
| **C. Unknown reason** | |
| 1) Principal Investigator or clinician should call patient to check in and conduct a survey to identify reason for non-compliance after patient misses 2 or more sessions; remind about the behavioral contract signed at the beginning of the study; repeat if needed if patient misses 2 more sessions after the check in call.  2) Medical reasons (medical appointments, TR conflicts with outpatient therapy, etc.)  3) Allow up to 2 missed sessions due to medical reasons; PI to check in if more than 2 sessions are missed due to medical reasons. | The PI identified that one of our participants lacked motivation to perform unsupervised sessions on his own. This helped us find a solution where the caregiver always helped motivate the patient start the unsupervised session as soon as she returned home from work. This helped improve compliance for this participant. |
| **D. Adverse events** | |
| In case of a related/unrelated adverse event that impacts TR participation, recommend consultation with the patient’s primary care provider and involving this provider in decision-making related to continuing TR therapy. Allow graded participant re-engagement if he/she can still complete 36 sessions in the 8-week period allowed. | One participant could not complete the scheduled sessions due to rotator cuff tear (occurring due to events unrelated to TR therapy) which was not formally diagnosed until after the allowable 8-week intervention period, and adverse event was reported and patient sessions were put on hold. |
| **IV. At home supervised** | |
| **A. Therapist unavailability (Unanticipated)** | |
| Allow therapists from other sites to supervise scheduled sessions, complying with HIPAA and organizational guidelines. |  |
| **V. Post-intervention assessment session (onsite)** | |
| **A. Transportation issue** | |
| Offer transportation to and from study site or offer to complete visit 2 (post intervention assessment visit) at patient's home during device pick up. | One of our participants had missed two scheduled visit 2 sessions. When further probed, she mentioned that she had transportation issues. Transportation was arranged to and from the study site for visit 2 and this visit was then completed. |
| **B. COVID-19 diagnosis** | |
| Reschedule based on CDC and organizational guidelines and complete follow-up as soon as safely recommended. | An enrolled participant was scheduled for visit 2 and when called to confirm the session a day before, reported that she tested positive for COVID-19. The research assistant was in contact with the participant after 5 days and continued to check in until she had a negative COVID test. Her visit 2 was rescheduled per study protocol guidelines (after 10 days of symptoms/negative test) as well as complying with institutional guidelines after clearance from the PIs. |

Table S2 lists strategies to effectively introduce telerehabilitation in the Inpatient Rehabilitation Facility and the home settings.
